# West Nile Virus Epizootiology, Central Red River Valley, North Dakota and Minnesota, 2002–2005

**DOI:** 10.3201/eid1208.060129

**Published:** 2006-08

**Authors:** Jeffrey A. Bell, Christina M. Brewer, Nathan J. Mickelson, Gabriel W. Garman, Jefferson A. Vaughan

**Affiliations:** * University of North Dakota, Grand Forks, North Dakota, USA

**Keywords:** West Nile virus, epitope-blocking enzyme-linked immunosorbent assay (ELISA), seroprevalence, passerine birds, *Culex tarsalis*, North Dakota, Minnesota, dispatch

## Abstract

West Nile virus (WNV) epizootiology was monitored from 2002 through 2005 in the area surrounding Grand Forks, North Dakota. Mosquitoes were tested for infection, and birds were surveyed for antibodies. In 2003, WNV was epidemic; in 2004, cool temperatures precluded WNV amplification; and in 2005, immunity in passerines decreased, but did not preclude, WNV amplification.

West Nile virus (WNV) is a flavivirus with an enzootic cycle that involves primarily mosquitoes and birds in the order Passeriformes. Since its introduction into the northern prairies of the United States in 2002, WNV has flourished. In 2003 and 2005, the prairie states of North Dakota, South Dakota, and Nebraska recorded the highest incidence of cases in humans (per 100,000 county residents) for the entire United States (*1*). Although WNV is still new to the region, the ecology of the northern prairie seems to offer favorable conditions for its continued enzootic transmission. This report chronicles the initial establishment of WNV within the central Red River Valley of eastern North Dakota and northwestern Minnesota (Figure).

**Figure:**
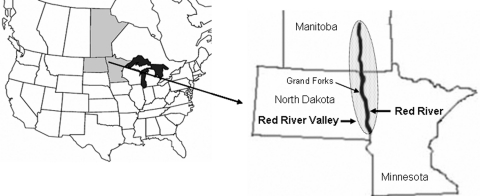
Red River Valley of North Dakota, Minnesota, and Manitoba.

## The Study

Host-seeking mosquitoes were collected in and around Grand Forks, North Dakota, by using Mosquito Magnet traps (American Biophysics Corp., North Kingston, RI, USA) for 4 transmission seasons, from early summer 2002 through fall 2005. Mosquitoes were sorted by species and tested for WNV by using reverse transcriptase PCR assays. WNV was detected only in *Culex tarsalis* (*2*).

Passerine birds in and around Grand Forks were surveyed for antibodies to WNV for 3 transmission seasons: June 24–October 27, 2003, April 4–July 7, 2004, and May 17–August 11, 2005. In 2003 and early 2004, birds were captured by using mist nets, blood (<0.1 mL) was obtained by brachial venipuncture, and birds were released. Later in 2004 and in 2005, necropsies were performed on dead birds. Blood spots were placed on filter paper and later eluted in 250 μL saline. Samples were tested for anti-WNV antibodies by using a qualitative epitope-blocking ELISA (*3*).

This is the first report of seroprevalence of WNV in passerines in the northern prairies. A total of 277 birds (11 species) were tested (Table 1). In 2003, seroprevalence was relatively low (17%). The first seropositive bird was captured July 24, 2003, 4 days after the first WNV-positive pool of *Cx. tarsalis* was detected (*2*). Most seropositive birds (11 of 14) were collected in September, when migratory species were leaving and vector populations were waning. Thus, a lag occurred between peak abundance of infected vectors in mid- to late August 2003 (*2*) and seroconversion of passerines. Seroprevalence rates were significantly higher in 2004 and 2005 than in 2003 (Table 1, Fisher exact tests, p<0.0001) and were higher than most seroprevalences reported for passerines in the eastern and southeastern regions of the United States (*4**–**6*). All passerine species sampled in 2004 and 2005 contained seropositive birds, which indicated that all these species were preyed on by vectors regardless of differences in their nesting habitats (e.g., cattail marshes, peridomestic). American robins, common grackles, and red-winged blackbirds showed increased seroprevalence from 2003 to 2004. High seroprevalence was maintained in passerines in 2005 despite low WNV activity (i.e., low natural boosting) during 2004 (*2*), which suggests that passerine immunity to WNV may last longer than a single season (*7**,**8*).

**Table 1:** Prevalence of antibodies against West Nile virus (WNV) in 11 species of passerine birds sampled within the central Red River Valley of North Dakota and Minnesota during 2003, 2004, and 2005

Common name	Scientific name	% birds with antibodies to WNV (n)		
		2003	2004	2005
American crow	*Corvus brachyrhynchos*	–	33 (6)	50 (6)
American robin	*Turdis migratorius*	18 (17)	50 (6)	38 (26)
Brown-headed cowbird	*Molothrus ater*	–	–	17 (6)
Blue jay	*Cyanocitta cristata*	50 (4)	–	87 (8)
Brewer's blackbird	*Euphagus cyanocephalus*	–	–	33 (3)
Common grackle	*Quiscalus quiscula*	0 (11)	71 (14)	63 (67)
Eastern kingbird	*Tyrannus tyrannus*	–	–	100 (3)
European starling	*Sturnus vulgaris*	–	100 (2)	67 (3)
Gray catbird	*Dumetella carolinensis*	–	75 (4)	–
House sparrow	*Passer domesticus*	20 (45)	–	50 (2)
Red-winged blackbird	*Agelaius phoeniceus*	0 (5)	50 (20)	63 (19)
Total		17.1 (82)	57.7 (52)	57.3 (143)

Surprisingly, American crows had a high seroprevalence to WNV. Previous laboratory and field studies have indicated that most American crows die so quickly from WNV infection that they never have time to seroconvert (*9**–**11*). Why crows in the Red River Valley survive WNV infection is not known. One possibility is that WNV has undergone genetic changes with a concurrent loss in virulence as it spread westward from forest ecosystems with *Cx. pipiens* with *Cx. restuans* as its primary vectors into prairie ecosystems and *Cx. tarsalis* as its primary vector (*2**,**12**,**13*).

Annual data on seroprevalence in passerines, environmental temperatures, reporting of human cases, and minimum infection rates (MIR) in vector populations are summarized in Table 2.

**Table 2:** Epizootiology of West Nile virus (WNV) within the central Red River Valley of North Dakota and Minnesota during the first 4 years of its introduction into the region*

Year	Primary transmission season†	Thermal accumulations (degree-days)‡	Vector abundance§	Human cases in ND¶	Seasonal MIR#	Passerine seroprevalence
2002, introductory	92 days (11 Jun–10 Sep)	1,067	230	17	0.0 (n = 5,871)	No birds tested
2003, epidemic	92 days (11 Jun–10 Sep)	1,022	21	617	5.7 (n = 5,432)	17% (n = 82)
2004, cold	51 days (7 Jul–1 Sep)	371	9	20	0.0 (n = 1,245)	58% (n = 52)
2005, equilibrium?	84 days (20 Jun–11 Sep)	867	29	86	1.3 (n = 3,123)	57% (n = 143)

*ND, North Dakota; MIR, minimum infection rate. †Time between first and last appearances of host-seeking *Culex tarsalis* mosquitoes in Mosquito Magnet traps. ‡Based on developmental threshold temperature of 14.3°C for WNV growth in *Cx. tarsalis* (*14*). §Average number of *Cx. tarsalis* mosquitoes captured per trap-night in Grand Forks, ND. ¶Data from North Dakota Department of Public Health (*15*). #No. of WNV-infected *Cx. tarsalis* mosquitoes per 1,000.

## Conclusions

Environmental conditions from 2002 to 2005 produced a natural field experiment, which demonstrated the differing magnitudes by which environmental temperature and host immunity affected local WNV activity. Despite warm temperatures and high vector abundance, WNV activity was low during its introductory year (2002), as indicated by low numbers of human cases, undetectably low seasonal MIR, and by low seroprevalence in passerines in 2003. Because WNV had only recently arrived, presumably there was neither sufficient time nor number of infection nidi to promote extensive amplification cycles. However, 2003 was an epidemic year for WNV, as indicated by the increased number of human cases statewide and the high MIR in the local vector population. The relatively low level of immunity in passerines at the time also likely contributed to the epidemic. In 2004, unusually cool environmental temperatures prolonged vector larval development, adult emergence, and the arboviral extrinsic incubation period. As a result, duration of the 2004 transmission season was nearly half that of the preceding seasons (Table 2). Thus, WNV activity during 2004 was low (i.e., reduced vector abundance, number of human cases, and seasonal MIR), and the virus had insufficient time to undergo extensive amplification cycles, similar to the situation that occurred during the introductory year of 2002.

However, the epidemic conditions of 2003 had produced a high level of herd immunity in the local bird population in 2004. This immunity carried over into 2005. (Note: most migratory passerine species live for several years and return each year to the same general locale to breed.) In 2005, environmental temperature, length of transmission season, and vector abundance were all nearly identical to those of the epidemic year of 2003. Yet the intensity of WNV activity during 2005 was considerably less than that during 2003. The big difference between 2003 and 2005 was the level of immunity in passerines. The high prevalence of immunity during 2005 may have contributed to preventing another epidemic, but it did not totally eliminate WNV activity. Levels of WNV activity during 2005 (as measured by human cases and mosquito MIR) were intermediate between those of the epidemic year (2003) and both the introductory year (2002) and the coldest year (2004). Thus, environmental temperature dictated the ultimate success (or more precisely, the failure) of WNV amplification within the central Red River Valley during 2004, whereas even high levels of herd immunity among the reservoir population exerted only a moderating effect on WNV activity during 2005.

Whether the level of WNV activity observed during 2005 will be representative of the arbovirus's natural equilibrium within the central Red River Valley remains to be seen. One uncertainty is how seasonal transmission is initiated. Is WNV reintroduced every spring through infected migratory birds or wind-blown mosquitoes from the south? Or does WNV survive the harsh winters inside mosquitoes undergoing diapause, only to reemerge in the spring? Either or both of these scenarios could be correct, but one conclusion is certain: the incidence of WNV disease in horses from North Dakota during June of 2002 and again in May of 2005 (*15*) indicates that WNV becomes active in the northern Great Plains well in advance of the first summer brood of its primary vector, *Cx. tarsalis* (*2*).
